# Supplementary figures and images for: Transcriptional regulation of cyclophilin D by BMP/Smad signaling and its role in osteogenic differentiation
Source: eLife. 2022 May 30;11:e75023. doi: 10.7554/eLife.75023 (PMC9191891; doi:10.7554/eLife.75023)

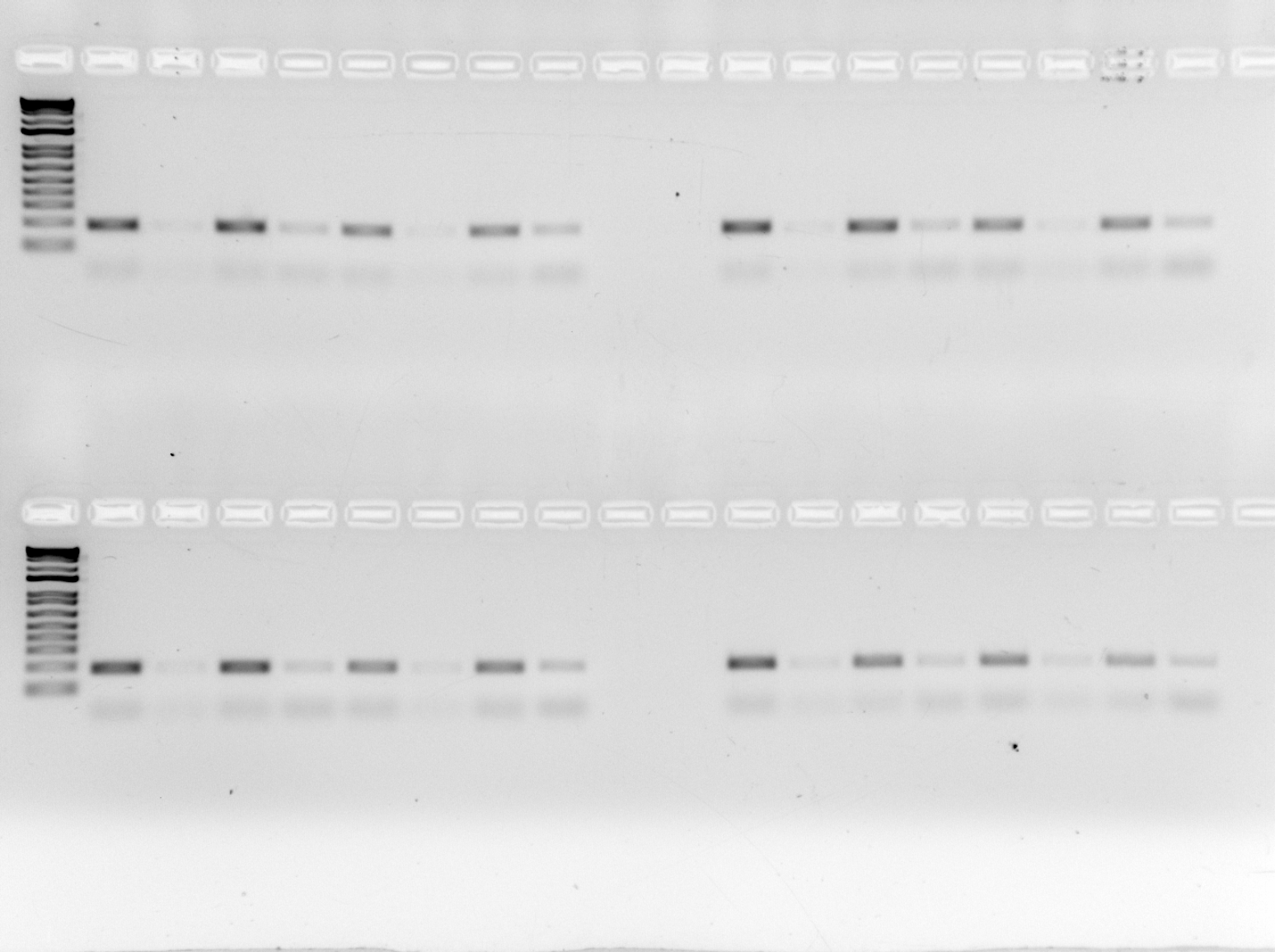

Supplement: Figure 2—source data 1. — PCR analysis of the ChIP assay was performed using primers to amplify the distal Smad-binding elements (SBE)-containing region within the Ppif promoter or the positive control Intron2 primers. Original gel image showing three independent experiments and an extra replicate. [file elife-75023-fig2-data1.zip › 931ff395-4862-42f1-b973-37a8309144db.tif]

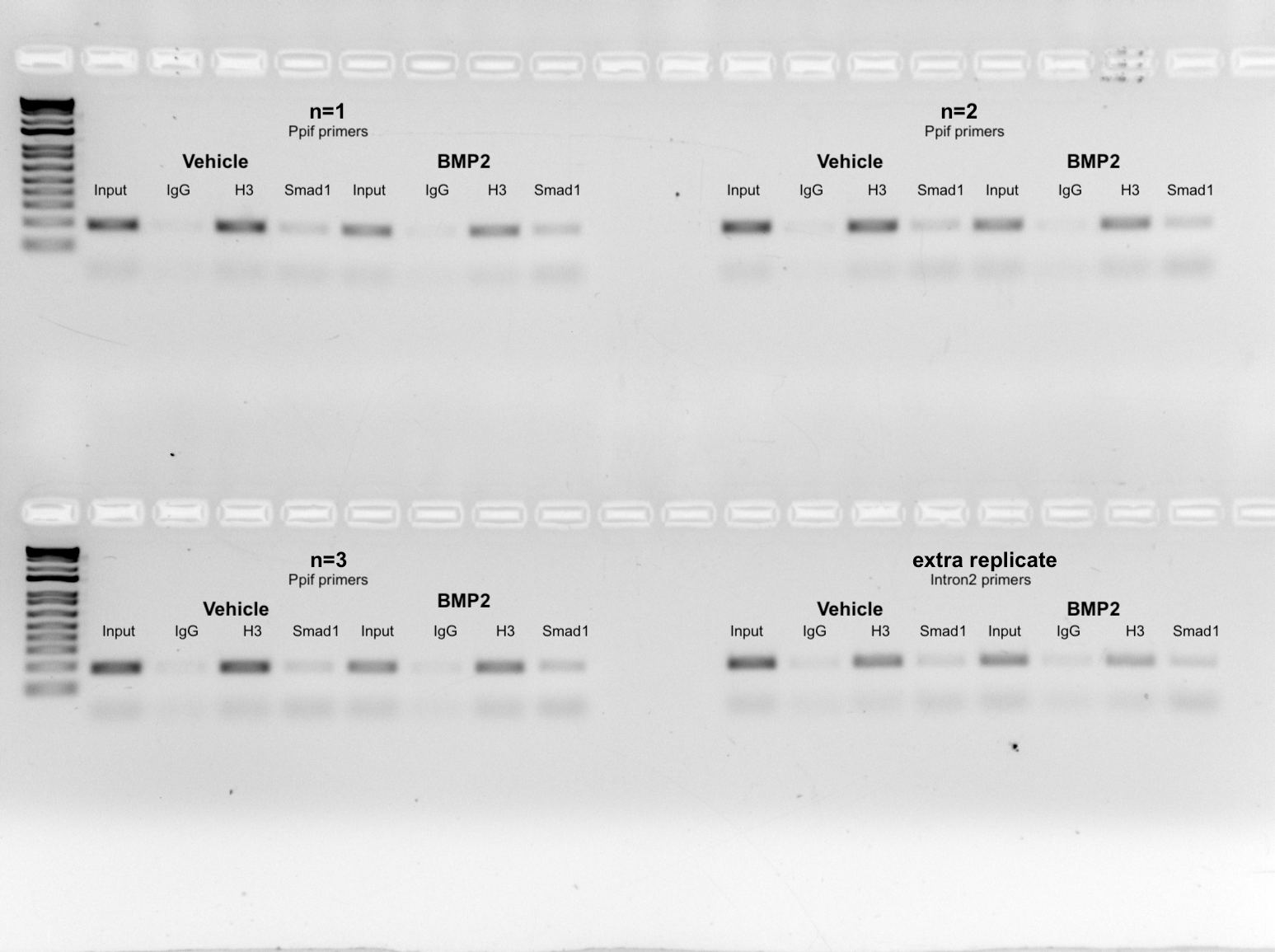

Supplement: Figure 2—source data 2. — PCR analysis of the ChIP assay was performed using primers to amplify the distal Smad-binding elements (SBE)-containing region within the Ppif promoter or the positive control Intron2 primers. Labeled gel image. Input: total DNA; IgG: negative control; H3: positive control; Smad1: Smad1 antibody. [file elife-75023-fig2-data2.zip › 0ba1aa66-c052-450f-9024-f17c6f0b618a.tif]

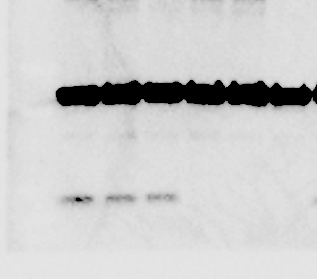

Supplement: Figure 4—figure supplement 2—source data 1. — Shown is the original western blot image probed for B-actin and cyclophilin D (CypD) (representative of 3). [file elife-75023-fig4-figsupp2-data1.zip › 21bfa228-adcb-439e-828d-51b8667d35c6.tif]

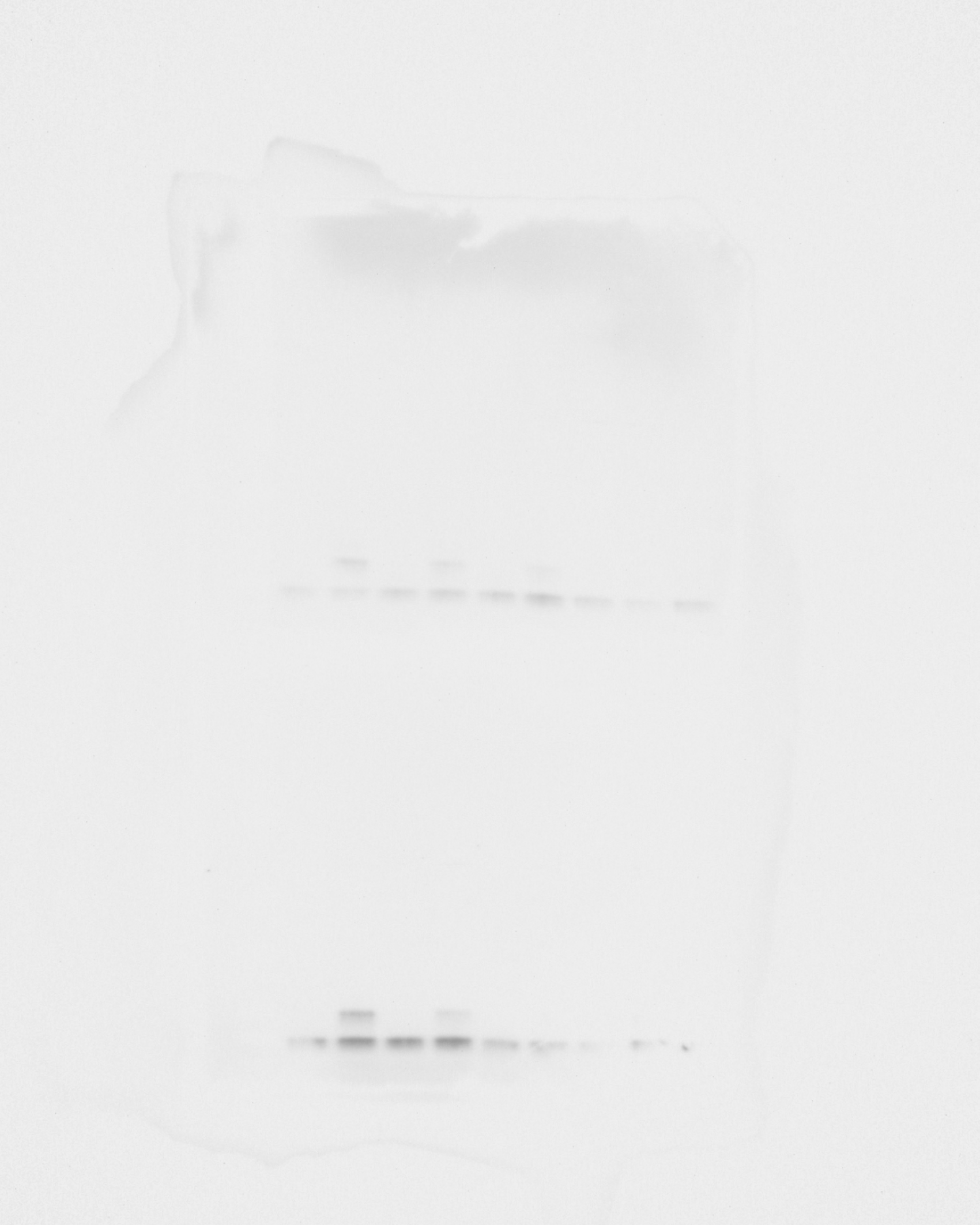

Supplement: Figure 6—source data 1. [file elife-75023-fig6-data1.zip › 077cd061-4e91-45d5-a757-e0a76bdc5676.tif]

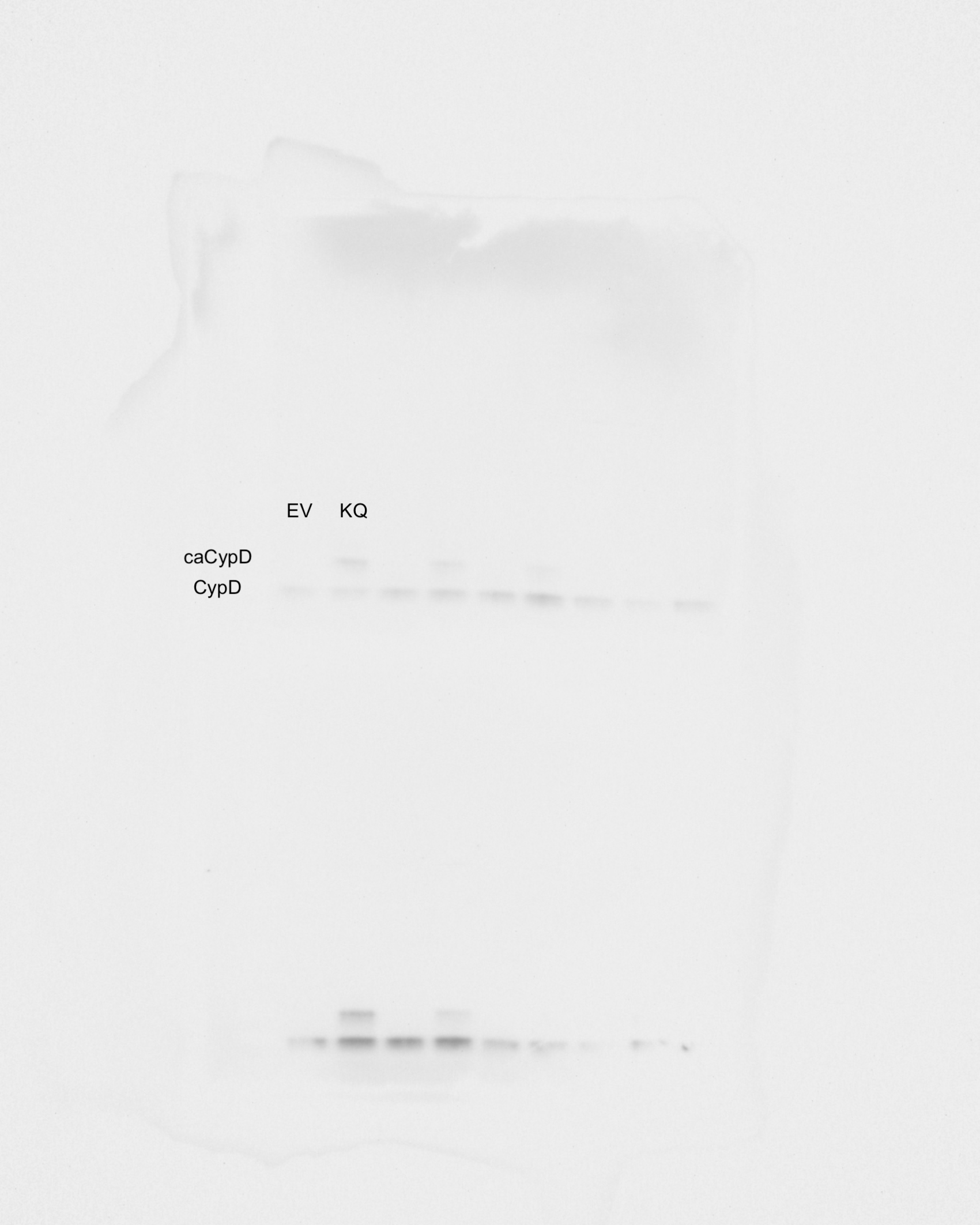

Supplement: Figure 6—source data 2. [file elife-75023-fig6-data2.zip › d1c2b2f1-550c-4482-abce-b202fd3ae4bb.tif]

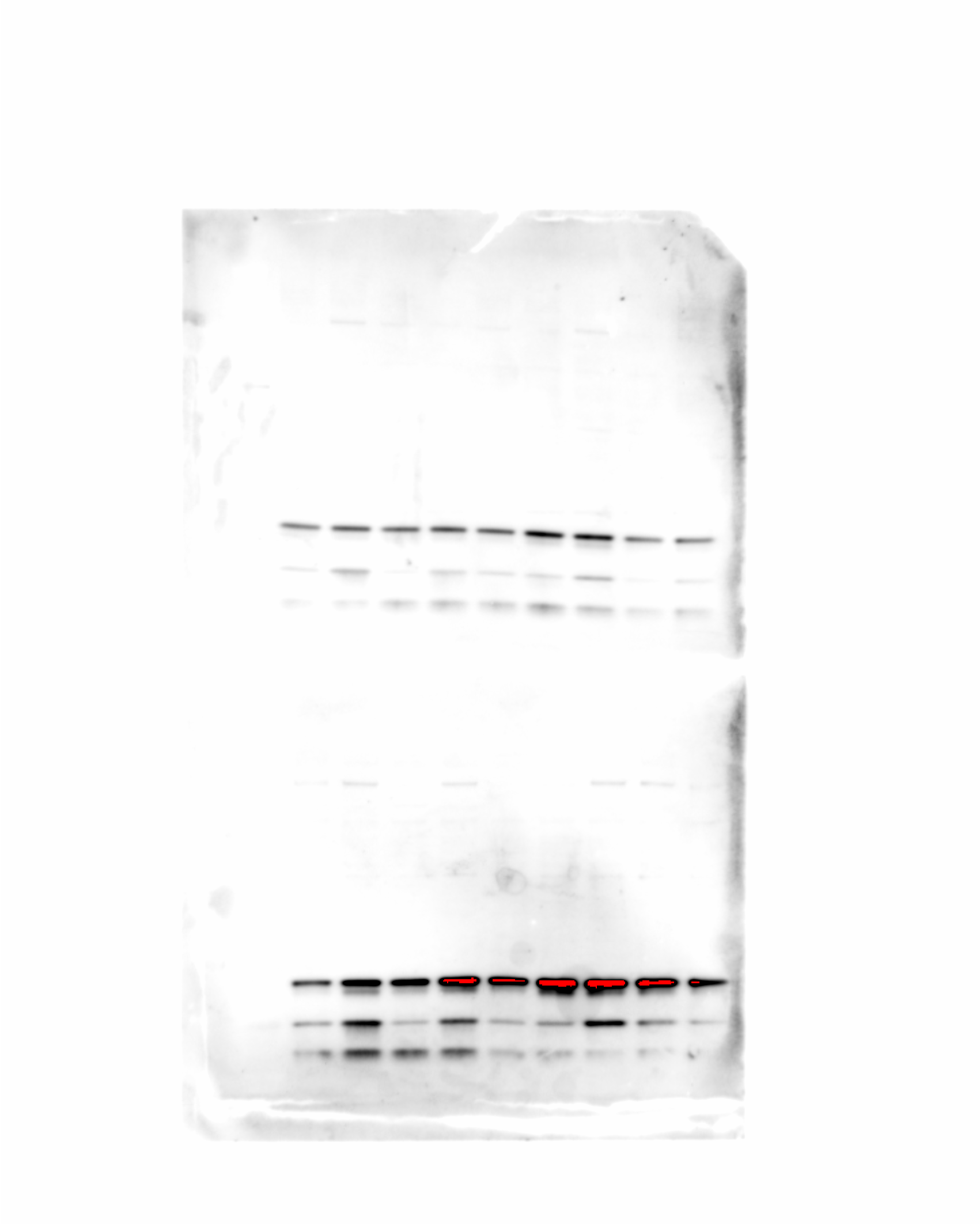

Supplement: Figure 6—source data 3. [file elife-75023-fig6-data3.zip › 22dcbf3e-0989-4ffb-ab07-fe3d60d1f95d.tif]

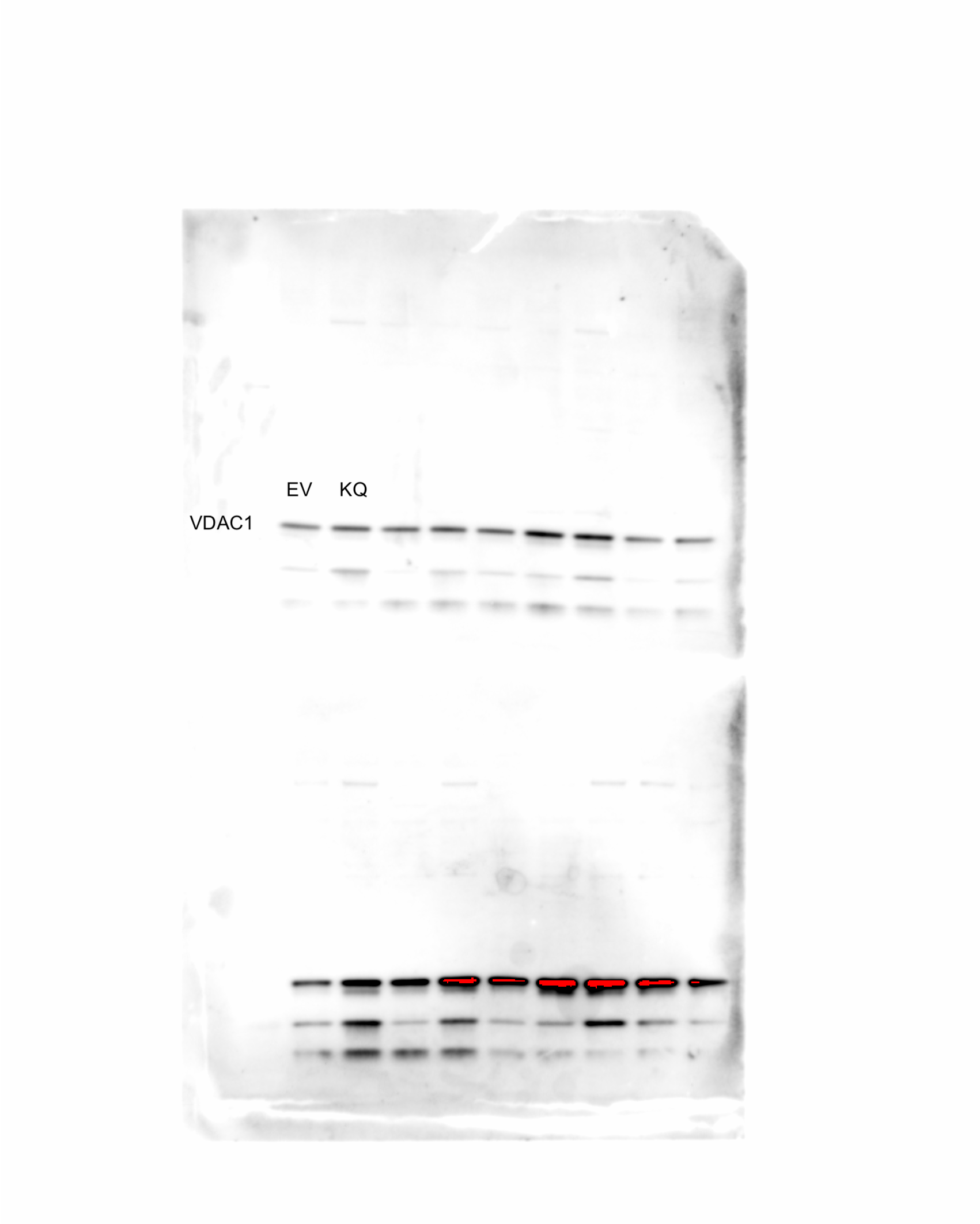

Supplement: Figure 6—source data 4. [file elife-75023-fig6-data4.zip › c59663ed-2f3f-4c71-92d1-f3d568559dee.tif]

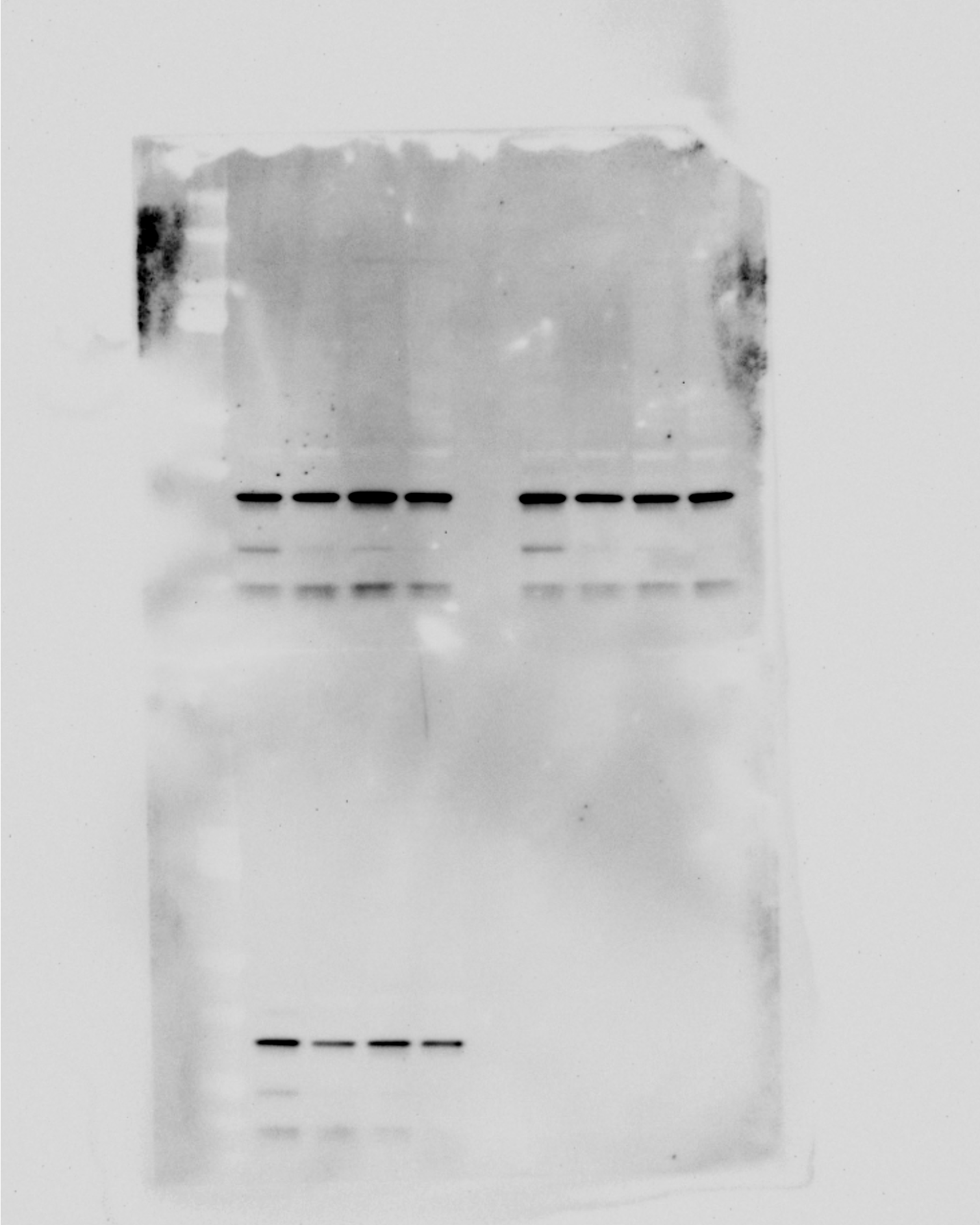

Supplement: Figure 6—source data 5. — (A) Original western blot image of BMSCs from 2.3 kb Col1CreERt2;R26caPpif/+ mice. [file elife-75023-fig6-data5.zip › 2e584d9a-4d1f-4c05-89e7-747265bd9349.tif]

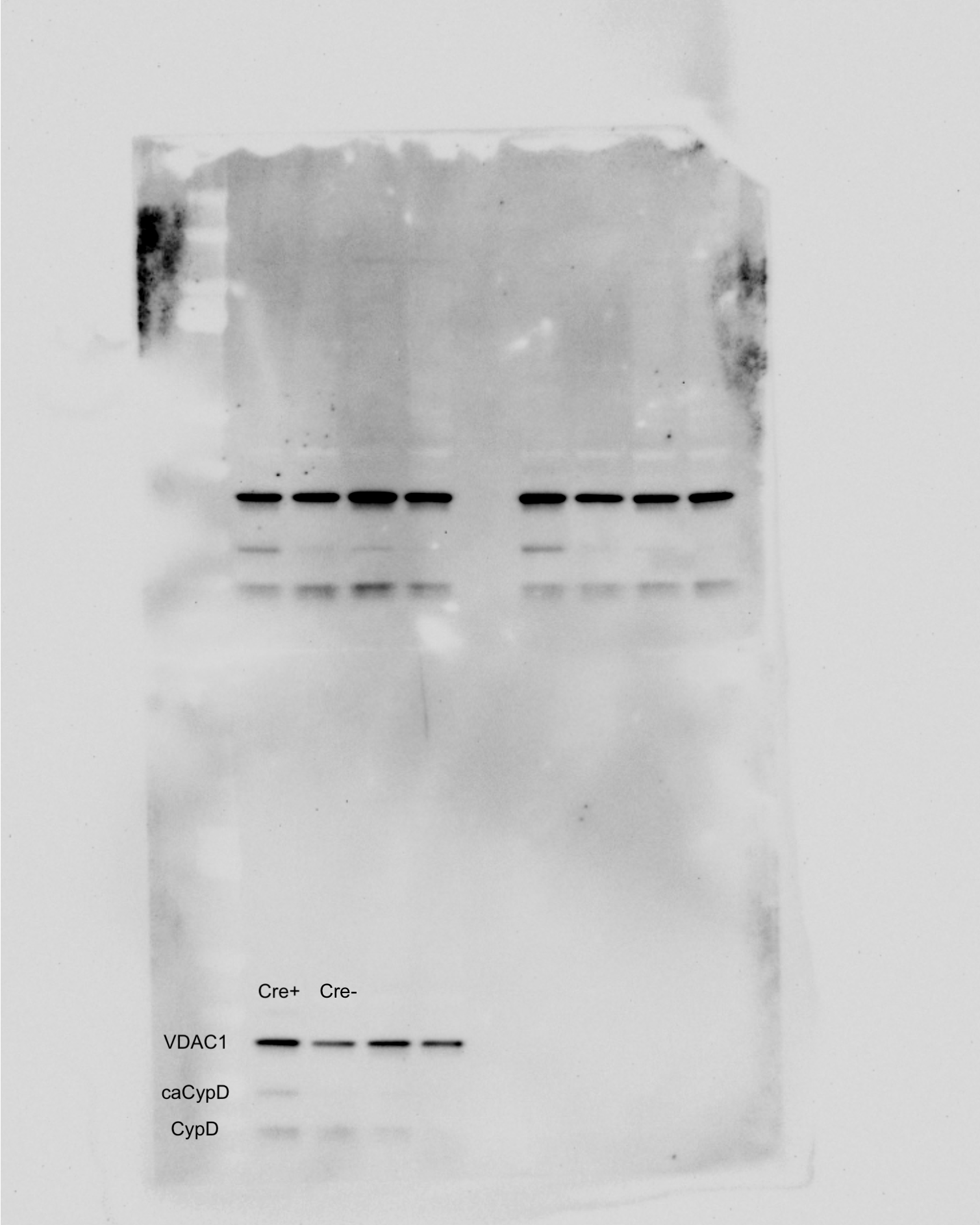

Supplement: Figure 6—source data 6. — (A) Labeled western blot image of BMSCs from 2.3 kb Col1CreERt2;R26caPpif/+ mice (VDAC1: loading control; caCypD: mutant CypD; CypD: endogenous CypD). [file elife-75023-fig6-data6.zip › 76fdf6c3-6212-46e3-8ede-274f02a37f6a.tif]

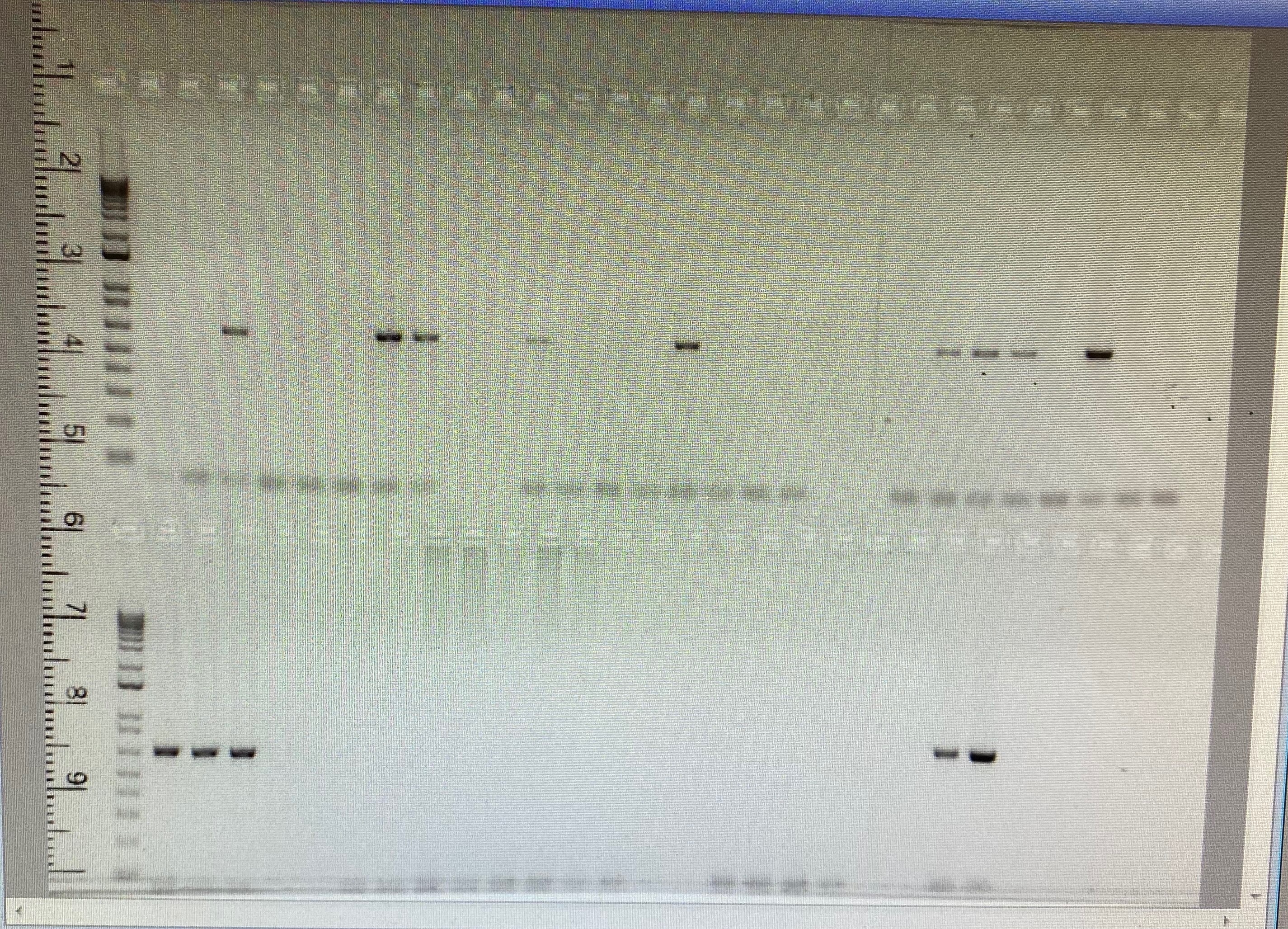

Supplement: Figure 6—figure supplement 1—source data 1. [file elife-75023-fig6-figsupp1-data1.zip › Figure 6-figure supplement 1-source data 1.jpg]

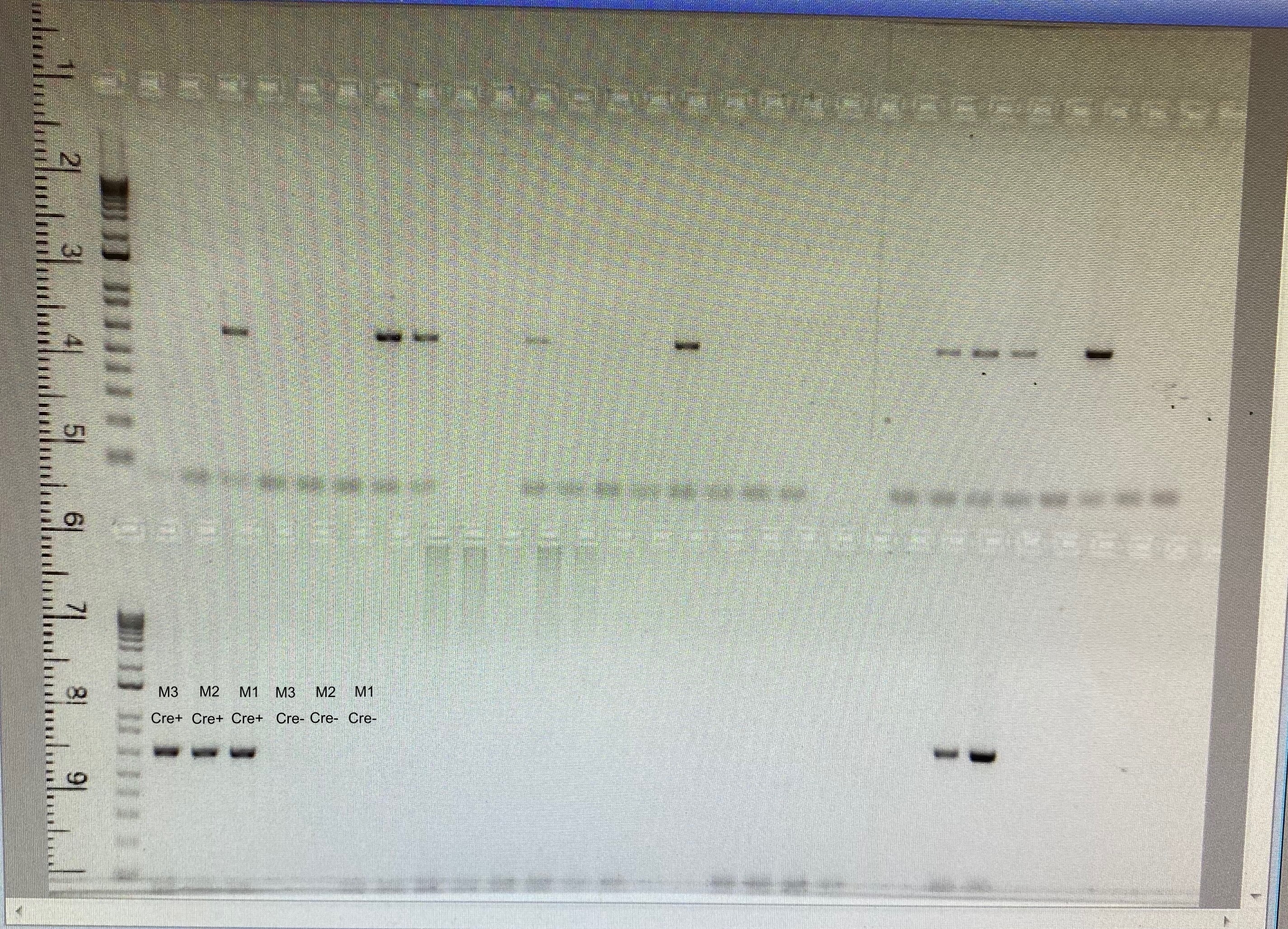

Supplement: Figure 6—figure supplement 1—source data 2. — Cre primer was used to allocate control and experimental groups. Labeled electrophoresed gel image shows DNA target amplified for Cre from three independent mice per group: Cre- control and Cre+ experimental mice. [file elife-75023-fig6-figsupp1-data2.zip › Figure 6-figure supplement 1-source data 2.jpg]

Bio-Plex 2019-12-10 14hr 10min

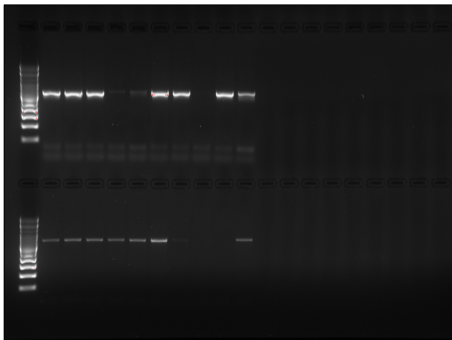

Supplement: Figure 6—figure supplement 1—source data 3. — Original electrophoresed gel image shows DNA target amplified for mutant CypD gene (caCypD) from three independent mice group: Cre- control and Cre+ experimental mice. [file elife-75023-fig6-figsupp1-data3.zip › Figure 6-figure supplement 1-source data 3.pdf]

Bio-Plex 2019-12-10 14hr 10min

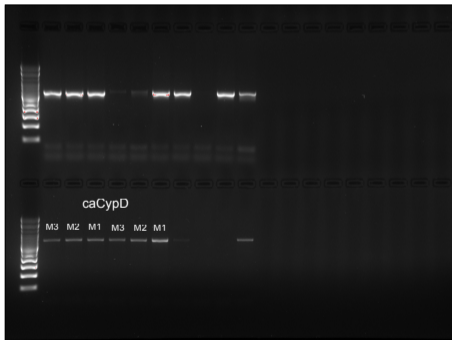

Supplement: Figure 6—figure supplement 1—source data 4. — Labeled electrophoresed gel image shows DNA target amplified for mutant CypD gene (caCypD) from three independent mice group: Cre- control and Cre+ experimental mice. [file elife-75023-fig6-figsupp1-data4.zip › Figure 6-figure supplement 1-source data 4.pdf]
